# Psychotherapy Access Barriers and Interest in Digital Mental Health Interventions Among Adults With Treatment Needs: Survey Study

**DOI:** 10.2196/65356

**Published:** 2025-04-01

**Authors:** Isabella Starvaggi, Lorenzo Lorenzo-Luaces

**Affiliations:** 1 Indiana University Bloomington Bloomington, IN United States

**Keywords:** psychotherapy, internet-based cognitive behavior therapy, internet-based interventions, guided self-help, public health, treatment access barriers, digital mental health, treatment attitudes

## Abstract

**Background:**

Digital mental health interventions (DMHIs) are a promising approach to reducing the public health burden of mental illness. DMHIs are efficacious, can provide evidence-based treatment with few resources, and are highly scalable relative to one-on-one face-to-face psychotherapy. There is potential for DMHIs to substantially reduce unmet treatment needs by circumventing structural barriers to treatment access (eg, cost, geography, and time). However, epidemiological research on perceived barriers to mental health care use demonstrates that attitudinal barriers, such as the lack of perceived need for treatment, are the most common self-reported reasons for not accessing care. Thus, the most important barriers to accessing traditional psychotherapy may also be barriers to accessing DMHIs.

**Objective:**

This study aimed to explore whether attitudinal barriers to traditional psychotherapy access might also serve as barriers to DMHI uptake. We explored the relationships between individuals’ structural versus attitudinal barriers to accessing psychotherapy and their indicators of potential use of internet-delivered guided self-help (GSH).

**Methods:**

We collected survey data from 971 US adults who were recruited online via Prolific and screened for the presence of psychological distress. Participants provided information about demographic characteristics, current symptoms, and the use of psychotherapy in the past year. Those without past-year psychotherapy use (640/971, 65.9%) answered questions about perceived barriers to psychotherapy access, selecting all contributing barriers to not using psychotherapy and a primary barrier. Participants also read detailed information about a GSH intervention. Primary outcomes were participants’ self-reported interest in the GSH intervention and self-reported likelihood of using the intervention if offered to them.

**Results:**

Individuals who had used psychotherapy in the past year reported greater interest in GSH than those who had not (odds ratio [OR] 2.38, 95% CI 1.86-3.06; *P*<.001) and greater self-reported likelihood of using GSH (OR 2.25, 95% CI 1.71-2.96; *P*<.001). Attitudinal primary barriers (eg, lack of perceived need; 336/640, 52.5%) were more common than structural primary barriers (eg, money or insurance; 244/640, 38.1%). Relative to endorsing a structural primary barrier, endorsing an attitudinal primary barrier was associated with lower interest in GSH (OR 0.44, 95% CI 0.32-0.6; across all 3 barrier types, *P*<.001) and lower self-reported likelihood of using GSH (OR 0.61, 95% CI 0.43-0.87; *P*=.045). We found no statistically significant differences in primary study outcomes by race or ethnicity or by income, but income had a statistically significant relationship with primary barrier type (ORs 0.27-3.71; *P*=.045).

**Conclusions:**

Our findings suggest that attitudinal barriers to traditional psychotherapy use may also serve as barriers to DMHI use, suggesting that disregarding the role of attitudinal barriers may limit the reach of DMHIs. Future research should seek to further understand the relationship between general treatment-seeking attitudes and attitudes about DMHIs to inform the design and marketing of DMHIs.

## Introduction

### Background

Over 50 million Americans experience mental illness in a given year [1,2], but only one-third of those diagnosed with a mental health condition receive treatment from a specialist mental health care provider [3]. Individuals with common mental disorders face many barriers to adequate care, including limited numbers of specialist mental health care providers, geographic barriers due to therapists living mostly in urban areas, and an inability to pay for or use insurance [3,4]. In recent years, there has been an increasing interest in the potential of low-cost, digitally delivered psychological treatments (eg, internet-based guided self-help [GSH] and smartphone apps) to provide treatment remotely and for low cost, such that they can be disseminated at a larger scale than “traditional” treatments, such as one-on-one face-to-face therapy [5]. These digital mental health interventions (DMHIs) may have the potential to expand access to effective psychological treatment [6] with a potentially revolutionary impact on public health, spurring a surge of attention across clinical research [7,8], the private sector [9,10], and government initiatives [11-13]. Robust evidence has now supported the efficacy of DMHIs in various formats, including internet-delivered cognitive behavioral therapy [14], both GSH and unguided self-help formats [14-16], and smartphone apps [16-18]. However, less research has focused on evaluating the potential *reach* of DMHIs [7,8].

Evidence suggests that public use of DMHIs may be lower than is required for broad public health impact. For example, DMHIs may be difficult for the public to access [19], underused by mental health care providers [20], or simpler and less appealing to the public than treatment developers might predict [21]. Therefore, it is vital that DMHI researchers afford sufficient attention to the actual implementation of DMHIs. Indeed, Ramos et al [7] cautioned that DMHI research risks mimicking the pitfalls of the “Decades of the Brain,” where large volumes of funding and time were spent on seemingly exciting innovations that made virtually no public health impact. Excellent work is underway to address this issue by identifying successful implementation strategies to maximize the reach of DMHIs, drawing from theoretical models and previous successes in implementation and dissemination science [7,22-24]. However, it may also be important to consider factors in individuals’ decisions to uptake and adopt DMHIs, drawing from literature on treatment-seeking behavior and mental health service use [25-28].

### Attitudinal Barriers to Treatment Access

The scale of impact of DMHIs is dependent on the extent to which individuals with unmet treatment needs are broadly *interested in* and *would use* DMHIs. Although DMHIs are designed to circumvent *structural barriers* to treatment access (eg, cost, geographic availability of mental health care providers, need for transportation, and time commitment), their reach may still be limited by *attitudinal barriers* to treatment uptake. Attitudinal barriers are beliefs held by individuals that may affect their treatment-seeking behavior, such as the lack of perceived need for treatment, stigma, beliefs about the efficacy of psychotherapy, and the desire to handle a problem on one’s own. Importantly, attitudinal barriers are at least as common as structural barriers [25-29]. For example, across 24 countries in the World Health Organization (WHO) World Mental Health surveys, only 38% of the individuals with a 12-month mental disorder diagnosis reported a perceived need for treatment, making low perceived need the most commonly reported barrier to treatment use [25]. Therefore, the most common barriers to accessing traditional treatment may not be addressed by the innovations of DMHIs, which could greatly limit their potential public health impact.

Because most common attitudinal barriers (eg, stigma) are likely to impact *any* form of mental health treatment seeking, it may be likely that barriers commonly reported for traditional psychotherapy will generalize to DMHIs. However, this has rarely been studied directly. A small, promising body of literature currently provides some information on attitudinal barriers that are specific and often unique to DMHIs. However, much of this research often focuses on the acceptability of using DMHIs [20,30], especially pertaining to issues with technology specifically rather than revealing the impact that attitudinal barriers to general mental health treatment seeking might have on DMHI uptake. In a 2021 systematic review, Borghouts et al [31] identified common barriers to user engagement with DMHIs across a variety of study types and DMHI formats (including telehealth treatment), in which information about barriers was often indirectly reported as secondary components of studies, such as clinical trials, or extracted from user reviews. Many of the reviewed studies investigated adherence and retention of participants receiving a DMHI rather than potential uptake among the general population. Across the studies, concerns specific to the use of technology were among the most commonly reported barriers for both participants and mental health care providers, such as privacy, digital literacy, and the ease of use. This literature advances our understanding of users’ experience of using DMHIs, which is essential for the design and dissemination of DMHIs that are acceptable and engaging from both user and provider perspectives. However, among 208 relevant articles, the authors identified only 5 studies that had assessed the relationship between participants’ broader mental health–related beliefs and engagement in DMHIs. For example, in a trial of a smartphone app for relationship stress among adolescents, perceived treatment needs and belief in treatment effectiveness were each associated with greater likelihood of use [32].

These findings provided some support for the idea that attitudinal barriers to mental health treatment seeking may generalize to DMHIs. However, none of these studies were conducted with the primary purpose of investigating this relationship. They did not gather comprehensive data surveying commonly experienced access barriers, as is typically done in studies of perceived barriers to mental health care [25-28]. Therefore, this work did not explore the relative impacts of reduced structural barriers versus remaining attitudinal barriers when considering the potential reach of DMHIs. More research is needed to understand the extent to which attitudinal and structural barriers to help seeking may limit the reach of DMHIs to individuals with unmet treatment needs.

### Low-Income Groups and Racial and Ethnic Minorities

In addition to hopes that DMHIs may expand treatment access in the general population, there is also an often-stated assumption that DMHIs have the potential to reduce racial, ethnic, and socioeconomic disparities in access to mental health care [33-35]. These groups are often underserved due to multifaceted, often systematic sets of access barriers. The relationship between income and actual treatment use may be more complex than is often assumed. For example, middle-income families may have lower access than low-income families due to the latter’s ability to use government-funded mental health services [36]. Nonetheless, the ability to pay for treatment or use insurance is one of the most commonly reported perceived barriers to mental health treatment seeking [5,25,26], especially in the United States [27]. In addition to the ability to pay [4], lower-income individuals face associated structural barriers, such as geographic restrictions, for example, specialty mental health care providers are twice as likely to be available in the highest-income communities relative to the lowest-income communities [37]. Socioeconomic disadvantages disproportionately impact racial and ethnic minorities, who are overrepresented in lower-income communities. In 2012, the rate of access to mental health care for non-Hispanic White American individuals was 20%, whereas the rates for Black, Hispanic, and Asian American individuals were 10%, 9%, and 5%, respectively [38]. It is very reasonable to assume that making DMHIs available can reduce these disparities by reducing the structural barriers that often contribute to them. However, only a limited body of research has collected data to substantiate this assumption, particularly in regard to racial and ethnic minorities [33], suggesting that the reach of DMHIs risks replicating the same inequities seen in traditional treatments [8,33,39].

It is essential to consider the role of attitudinal barriers in the ability of DMHIs to ameliorate mental health care disparities, given their great influence on treatment use in the general population. First, the impact of DMHIs on mental health disparities may be limited by attitudinal barriers because attitudinal barriers can limit the extent to which services are used even if they are made accessible. Second, some literature suggests that certain attitudinal barriers may disproportionately affect underserved groups, raising the concern that targeting disparities via the dissemination of DMHIs may disregard potential contributions of attitudinal barriers as an important mechanism of these disparities. For example, some studies suggest that individuals with lower incomes and lower educational attainment have a lower perceived need for treatment than individuals with higher incomes [40]. Similar findings regarding racial and ethnic minorities are mixed, partially because attitudes vary both across different minority groups and intersectionally within them. For example, Asian American and Black American individuals report lower perceived need for treatment than non-Hispanic White American individuals, but Hispanic American individuals report approximately the same level of need as non-Hispanic White American individuals [41]. This picture is further complicated by intersectional differences. For example, despite those group-level differences, Hispanic men report *greater* need for treatment than non-Hispanic White men, and Black women report approximately the same level of need as non-Hispanic White men [41]. Cultural beliefs about mental health treatment seeking also vary by immigration status (immigrant vs US born) and country of origin, often, despite similar racial identities [40,41].

Existing literature exploring racial differences in perceived barriers to treatment use is severely limited [31]. Some data exist regarding racial differences in interest in DMHIs. For example, Hispanic adults may be more interested in DMHIs than non-Hispanic White adults [34,42-44]; however, researchers have suggested this may be attributable to greater rates of smartphone use rather than greater willingness to use treatment overall. Unfortunately, there is limited data to further clarify these patterns because individuals from both lower-income and racial and ethnic minoritized groups are often left out of DMHI trials, such that their preferences and attitudes may be overlooked in the design delivery and content of DMHIs [33,34,39,45]. Neglecting to understand actual interest in DMHIs among underserved and marginalized groups may limit the potential of DMHIs to attract members of these groups and serve their mental health needs [33,34,46]. Because attitudinal barriers are such a significant deterrent to treatment seeking and treatment access, paying special attention to how attitudinal barriers may translate to DMHIs in these groups will be key to ensuring DMHIs reach them as intended.

### This Study

Existing literature has focused on developing innovative approaches to address structural barriers (ie, DMHIs), leaving a substantial gap in the effort to address attitudinal barriers. Therefore, it is unclear to what extent attitudinal barriers that limit access to traditional psychotherapy might generalize to also limit access to DMHIs, reducing the potential reach of DMHIs to populations with unmet treatment needs. In this study, we aimed to conduct an exploratory investigation of the relationship between attitudinal barriers to traditional psychotherapy access and potential use of DMHIs. We placed an emphasis on currently underserved populations such as racial and ethnic minorities and low-income groups in order to focus on those whom DMHIs are most intended to target. We captured both participants’ *interest* in GSH and their self-reported likelihood of *using* it. This allows us to parse the appeal of the intervention from its actual potential to engage participants. Research applying the theory of planned behavior [47] supports the use of behavioral intentions as a reliable predictor of mental health help seeking [48,49], and behavioral estimates may be an even stronger predictor than intentions [50].

## Methods

### Ethical Considerations

Study procedures were approved by the Indiana University Human Subjects and Institutional Review Board (#14172). Participants were provided with an informed consent document with information about our laboratory, the study’s purposes and procedures, payment, risks and benefits, confidentiality and data security, and their right to withdraw consent at any time. The document was written at an approximately seventh-grade reading level to ensure accessibility across differing literacy or English fluency. Participants were required to attest that they were aged ≥18 years before proceeding. Participants were paid at a rate of approximately US $10 per hour (US $3.02 total for a median time expenditure of 17 minutes and 59 seconds; IQR 13 minutes 53 seconds to 24 minutes and 18 seconds). First, we chose Prolific over other platforms such as Amazon Mechanical Turk (MTurk) due to its stated commitment to ethical treatment of its online workers, including its “ethical reward” payment policy [51-53]. Second, we designed our study to address established ethical considerations in DMHI research. For example, we used liberal inclusion and exclusion criteria in order to capture a broad population of individuals with treatment needs (eg, no suicidality exclusion) [54,55]. Finally, online DMHI research must carefully consider clinical risk monitoring [56]. We provided participants with crisis resources for suicidality as well as information about how to find noncrisis treatment and direct access to a free online self-help booklet (refer to the subsequent sections) [57].

### Web-Based Data Collection

#### Data Collection Platform

Participants were US adults recruited via the web-based crowdsourcing data collection platform Prolific (accessed from July 25to July 26, 2022) [58]. We chose Prolific because it is designed to improve upon common problems in internet-based human subjects research with crowdsourcing platforms, such as “bots” [59], the lack of engagement, repeat responders, and nongenuine responses, which can create serious issues in the quality of health research [60]. In this regard, Prolific’s quality control is superior to other platforms such as MTurk. Studies show that responders from Prolific are mostly rated as “high quality,” significantly outperforming MTurk and even undergraduate students [61,62].

#### Data Quality Checks

We used a series of methods, informed by previous literature, to reduce the risk that “fraudsters” and low-quality responders would pose a threat to the integrity of the data [59,60]. First, participants were required to pass a reCAPTCHA task (version 1; Google LLC) [63]. Second, participants completed a series of reading comprehension questions designed to screen out nonhuman or inattentive responders (refer to the Survey Procedure section). Participants who answered >1 question wrong after 2 attempts were eliminated in the data cleaning process. After data collection, we also removed responders who completed the survey too quickly (ie, 2 SDs below the mean completion time).

### Survey Procedure

#### Overview

Participants provided the data for this study in 2 separate surveys, which is the procedure required in order to implement a nondemographic eligibility requirement on Prolific. The first survey was primarily for screening purposes but also included several demographic characteristics, a measure of psychological distress used as the screening criterion (the Kessler-6 Psychological Distress Scale [K6]; refer to the Measures and Variables section [64]), and 2 other brief self-report measures not used in the present analyses. Participants who met the K6 eligibility criterion (scoring >5) were invited to complete the second survey, which included the following: (1) further demographic characteristics, (2) a description of a particular GSH intervention described in the subsequent sections, (3) questions about their past-year mental health treatment use, and (4) several other self-report measures not analyzed in this study.

#### GSH Intervention Description and Questions

Participants were provided information regarding a transdiagnostic GSH, Doing What Matters in Times of Stress (DWM), as it is delivered in clinical trials run by our laboratory. In the DWM intervention, trial participants are provided with a booklet developed by the WHO [57] that teaches principles and skills from acceptance and commitment therapy (ACT) [65]. They are provided the option to either access the booklet online (via a publicly posted PDF on the WHO website) or receive a paper copy in the mail. Guidance is provided by “coaches” (trained research assistants and graduate students in our laboratory), who meet with trial participants weekly via Zoom (Zoom Communications Inc) for 15- to 30-minute sessions across 3 to 6 weeks [66,67]. All survey participants were shown an advertisement used in our laboratory’s clinical trials and an additional description of basic information about DWM.

Participants were then asked to predict their likelihood of using the DWM intervention in the following series of questions:

- First, participants were asked if they believe that they would click on the advertisement and complete a 15-minute survey if they saw it on social media (“Do you think you would click on the link in the ad...”; “Most likely yes” or “Most likely no”).
- Those who answered “Most likely yes” to the aforementioned question were asked if they believed that they would then answer a call from a researcher and stay on the phone for approximately 30 minutes (“Do you think you would answer the call...”; “Most likely yes” or “Most likely no”).
- Those who answered “Most likely yes” to the phone call question were provided information about the trial. Those who answered “Most likely no” were provided the same information before rating their interest in the intervention later in the survey. All participants were required to pass a set of comprehension questions.
- After learning the information typically shared in the trial’s welcome call, participants were asked if they believed they would be likely to enroll in the intervention portion of the trial (“Do you think you would sign up for the treatment?”; “Most likely yes” or “Most likely no”).
- Those who answered “Most likely yes” were asked how many GSH sessions they believed they would attend (“How long do you think you would most likely stay in the treatment [how many weeks of calls with the coach do you think you would do]?”; “I would probably not attend any of the sessions” or “I would probably do the first few calls with the coach [1 to 2 weeks] but not finish all the ones I scheduled” or “I would probably do all the calls with the coach that I signed up for [3 to 6 weeks]”).

### Measures and Variables

#### Psychological Distress

Psychological distress was measured via the K6 [64], a 6-item measure assessing frequency of emotional distress over the past 30 days (eg, “During the past 30 days, about how often did you feel...Nervous,” “...Hopeless,” “...Worthless”), where higher scores indicate greater distress. The K6 is a reliable and valid measure of distress [68,69] that has been reported to have excellent internal consistency in previous work (Cronbach α=0.89) [70]. It appeared internally consistent in this study (ω=0.79). The K6 can be used to screen for both serious mental illness at a score of ≥13 [64] and milder forms of emotional distress with lower thresholds [71]. While investigators differ in describing the lower cutoffs as mild, moderate, or mild moderate, evidence generally supports the interpretation of a score >5 as indicating at least mild mental health needs [66,67,71].

#### Demographic Characteristics

Participants answered questions about age, race, ethnicity, gender, sex, sexual orientation, income, and education level (refer to the Results section).

#### Self-Reported Likelihood of GSH Use

After reading about the GSH intervention, participants answered a series of questions about their hypothetical use of the intervention. The *self-reported likelihood of GSH use* or “likely GSH use” outcome reflects endorsement that a participant believes that they would be likely to complete at least 1 GSH session if offered the intervention (ie, either “I would probably do the first few calls with the coach [1 to 2 weeks] but not finish all the ones I scheduled” or “I would probably do all the calls I signed up for”). Participants who did not reach this question due to denying that they believed they were likely to click on the advertisement, answer the phone call, or agree to enroll in the trial were coded as deniers of likely GSH use.

#### Interest in GSH

After answering the GSH use questions, participants were asked to rate their overall interest in the intervention on a 4-point scale (“Overall, does this treatment sound like something you would be interested in?”; “Not at all interested,” “Somewhat interested,” “Moderately interested,” or “Very interested”). The ordinal value on this 4-point scale is the *GSH interest* outcome.

#### Past-Year Psychotherapy Use

Following the survey section regarding the GSH intervention, participants proceeded to a survey section focusing on their actual past-year mental health treatment experiences. Participants indicated whether they had attended psychotherapy in the past year (“I went to therapy: seeing a mental health professional such as a psychologist, counselor, therapist, or social worker”) from a checklist of various forms of help (medications, informal social support, self-help, and other).

#### Perceived Need for Psychotherapy

Participants who denied any past-year psychotherapy use were asked whether there was any point in the past year at which they “thought [they] might benefit” from psychotherapy (“Yes, therapy: seeing a mental health professional like a counselor, psychologist, or social worker”), in the same checklist format as mentioned earlier. Selecting this answer choice was considered an endorsement of the *perceived need for psychotherapy* barrier; leaving it blank was considered to indicate the *lack of perceived need* barrier.

#### Barriers to Psychotherapy Use

Individuals who endorsed perceived need for psychotherapy were then presented with questions regarding their reasons for not receiving psychotherapy despite believing that they might need it. They were asked to select all contributing barriers for not accessing psychotherapy (“Please check *all* reasons that were part of why you did not go to therapy”). The answer choices included 11 common barriers to mental health treatment adapted from the National Comorbidity Survey [25,29]. A list of the 11 barriers is given in the Results section. The full text of each answer choice is provided in Multimedia Appendix 1. Text entry responses under “other” were manually coded to either match an existing answer choice (eg, “don’t have the money” into the “money/insurance” answer choice) or remain in the “other” category if no obvious match was present.

#### Primary Barrier and Primary Barrier Type

After selecting all barriers that contributed to their lack of past-year psychotherapy use, participants were presented with the same list of barriers and asked to indicate their *primary* barrier for not accessing psychotherapy (“Which was the *biggest* reason you didn’t go to therapy?”). We generated a *primary barrier type* variable by grouping individual primary barrier choices into 3 categories according to previous literature [25-28] and the authors’ judgment: attitudinal (eg, “Didn’t think it would work”), structural (eg, “Issues with money or health insurance”), and other (eg, “The problem went away by itself”). Denial of *perceived need for psychotherapy* was considered an attitudinal primary barrier. When participants selected “other” as their primary barrier, the text that they entered was coded into one of the existing categories for *primary barrier type.* They were recoded to be (1) *attitudinal* (eg, “too much history. how could I even begin to get a new person up to [speed]”), (2) *structural* (eg, “no privacy at home guaranteed for phone appointment”), or (3) *other* if did not clearly fit either category (eg, “anxiety”).

### Analyses

All analyses were performed in R software (version 4.3.2; R Foundation for Statistical Computing) [72]. First, we generated descriptive statistics for sociodemographic characteristics, psychological distress severity, and each of the primary outcomes (GSH interest and likely GSH use). We analyzed the relationship of individual characteristics (sociodemographic characteristics and psychological distress) with GSH interest via multivariate polychoric regression in the *MASS* package (version 7.3-60) [73] and the relationship of these predictors with likely GSH use via multivariate logistic regression. For all analyses involving income as a predictor, 1 participant was omitted due to a missing value for income. We additionally conducted univariate analyses for race and income, in order to isolate these key sociodemographic variables emphasized in the DMHI literature via 2 univariate polychoric regressions for GSH interest and 2 univariate logistic regressions for likely GSH use. These univariate analyses were informative in addition to the multivariate analyses of sociodemographic characteristics because we were substantively interested in the potential for DMHIs to reach low-income groups and marginalized racial-ethnic groups regardless of whether other demographic characteristics (eg, education) account for the difference.

Next, we generated descriptives for past-year psychotherapy use and analyzed its relationship with individual characteristics via logistic regression. We analyzed past-year psychotherapy use as a predictor of each GSH interest and likely GSH use via polychoric regression and logistic regression, respectively, controlling for psychological distress in both analyses. For all polychoric regressions, *P* values are not reported because the analysis does not directly generate *P* values, and simulated *P* values may not be reliable; our interpretations of statistical significance were made from CIs. Next, for participants who denied past-year psychotherapy use, we generated frequency statistics regarding endorsement of (1) perceived need for psychotherapy, (2) all contributing barriers to psychotherapy use, (3) primary barrier to psychotherapy use, and (4) primary barrier type. We only compared the frequency of lacking perceived need versus other *primary* barriers, rather than versus the frequency of all contributing barriers, due to the structure of our survey. Because participants who denied perceived need were not given the opportunity to select additional barriers, the level of detail collected about this subgroup’s access barriers was lower than for the subgroup that selected multiple contributing barriers.

Finally, we grouped the primary barriers into 3 categories: attitudinal, structural, and “other” (refer to the Primary Access Barriers and Barrier Type section). We analyzed sociodemographic characteristics and psychological distress as predictors of primary barrier type via multinomial regression in the *nnet* package (version 7.3-19) [73] to accommodate the 3-category outcome. Next, we analyzed primary barrier type as a predictor of each GSH interest and likely GSH use via polychoric regression and logistic regression, respectively, controlling for psychological distress in both analyses. Finally, we analyzed the relationship between each contributing barrier and each outcome via a series of univariate linear regressions.

Due to the high number of statistical tests, we applied Benjamini-Hochberg adjustment to *P* values across all analyses.

### Transparency and Openness

We report on how we determined our sample, all data exclusions, all manipulations, and all measures in the study. All data, analysis code, and research materials are available on the Open Science Framework website online [74]. This study’s design and its analysis were not preregistered. No other papers currently use these data.

## Results

### Descriptives and Sociodemographic Characteristics

Most of the 971 participants identified as non-Hispanic White (n=665, 68.5%) and heterosexual (n=688, 70.9%), with an approximately even gender split (women: n=538, 55.4%) and a median age of 32 (IQR 25-41) years. The median K6 score was 11 (IQR 8-15) of 0 to 24 possible points. Full sample characteristics are reported in Table 1.

**Table 1:** Sociodemographic characteristics for a sample of adults with psychological distress (N=971).

Variable			Values
Age (years), median (IQR)			32 (25-41)
**Gender, n (%)**			
	Man	390 (40.2)	
	Woman	538 (55.4)	
	Nonbinary, other identity, or undisclosed	43 (4.4)	
**Sexual orientation, n (%)**			
	Straight	688 (70.9)	
	Gay or lesbian	62 (6.4)	
	Bisexual	179 (18.4)	
	Other or undisclosed	42 (4.3)	
**Race and ethnicity, n (%)**			
	Asian	85 (8.8)	
	Hispanic	105 (10.8)	
	Non-Hispanic Black	63 (6.5)	
	Non-Hispanic White	665 (68.5)	
	Other or multiracial	53 (5.5)	
**Income (US $), n (%)**			
	<15,000	122 (12.6)	
	15,000-25,000	101 (10.4)	
	25,000-34,999	116 (12)	
	35,000-49,999	145 (15)	
	50,000-74,999	191 (19.7)	
	75,000-99,999	122 (12.6)	
	100,000-149,999	111 (11.4)	
	>150,000	62 (6.4)	
**Highest educational attainment, n (%)**			
	High school diploma or lower	142 (14.6)	
	No college	257 (26.5)	
	Associate’s degree	92 (9.5)	
	Bachelor’s degree	354 (36.5)	
	Graduate degree	126 (13)	
Psychological distress (K6^a^: 0-24), median (IQR)			11 (7)

^a^K6: Kessler-6 Psychological Distress Scale.

Most of the 971 participants (n=782, 80.5%) reported that they were at least “somewhat interested” in GSH. Nearly half (n=458, 47.2%) of the participants were at least “moderately interested,” and 17.1% (n=166) were “very interested.” However, a slightly greater proportion (n=189, 19.5%) of participants was “not at all interested.” We found that 38.6% (n=375) of the participants reported that they believed they were likely to complete at least one GSH session if it were offered to them.

### Individual Characteristics as Predictors of GSH Interest and Self-Reported Likelihood of GSH Use

None of the sociodemographic characteristics, including psychological distress, were statistically significant predictors of self-reported interest in GSH. For self-reported likelihood of GSH use, only age met statistical significance at *P*<.05 after Benjamini-Hochberg adjustment (odds ratio [OR] 1.02, 95% CI 1-1.03; *P*=.045). Table 2 gives full results for both outcomes.

**Table 2:** Results of 2 multivariate regression models: (1) polychoric regression predicting interest in guided self-help and (2) logistic regression predicting self-reported likelihood of using guided self-help, each from sociodemographic characteristics in adults with psychological distress (n=970a).

Variable		Interest in GSH^b^			Self-reported likelihood of GSH use^c^			
		OR^d^ (95% CI)	Adjusted *P* value^e,f^		OR (95% CI)		Adjusted *P* value^e,f^	
Age		1.01 (1.00-1.02)	.30		1.02 (1.00-1.03)		.045	
**Gender**				.60				.59
	Men	—^g^			—			
	Women	1.16 (0.91-1.49)			0.89 (0.67-1.18)			
	Nonbinary, other identity, or undisclosed	1.33 (0.72-2.45)			1.35 (0.67-2.72)			
**Race and ethnicity**				.72				.55
	Non-Hispanic White	—			—			
	Asian	0.79 (0.52-1.19)			0.60 (0.34-1.00)			
	Hispanic	1.24 (0.84-1.82)			1.06 (0.68-1.64)			
	Non-Hispanic Black	1.13 (0.72-1.77)			1.01 (0.58-1.75)			
	Other or multiracial	1.00 (0.60-1.66)			1.19 (0.66-2.14)			
**Sexual orientation**				.19				.81
	Straight	—			—			
	Gay or lesbian	1.47 (0.91-2.39)			0.97 (0.55-1.68)			
	Bisexual	1.21 (0.87-1.67)			1.24 (0.85-1.79)			
	Other or undisclosed	0.59 (0.32-1.08)			0.86 (0.41-1.73)			
**Income (US $)**				.43				.13
	<15,000	—			—			
	15,000-25,000	0.87 (0.58-1.30)			1.31 (0.82-2.08)			
	25,000-34,999	0.85 (0.59-1.21)			0.82 (0.54-1.24)			
	35,000-49,999	0.90 (0.63-1.28)			1.13 (0.75-1.70)			
	50,000-74,999	0.67 (0.47-0.94)			0.72 (0.49-1.07)			
	75,000-99,999	0.94 (0.67-1.31)			1.24 (0.85-1.81)			
	100,000-149,999	1.33 (0.96-1.84)			1.66 (1.15-2.39)			
	>150,000	1.02 (0.76-1.36)			0.87 (0.62-1.22)			
**Education**				.15				.83
	No college	—			—			
	Some college	1.44 (1.05-1.97)			1.17 (0.81-1.68)			
	Associate’s degree	0.84 (0.60-1.15)			1.12 (0.78-1.63)			
	Bachelor’s degree	1.09 (0.86-1.37)			1.17 (0.90-1.53)			
	Graduate degree	0.80 (0.59-1.10)			0.87 (0.61-1.24)			
Psychological distress (K6^h^: 0-24)		1.03 (1.00-1.06)	.12		1.02 (0.98-1.05)		.58	

^a^Each model is based on 970 participants with complete demographic data because 1 participant had a missing value for income.

^b^GSH: guided self-help.

^c^The reference level is denying the likely use of GSH, such that odds ratio >1 reflects an association with endorsing the likely use of GSH.

^d^OR: odds ratio.

^e^For categorical variables (all except age and psychological distress), the *P* value shown is an omnibus *P* value calculated across levels of the variable.

^f^Benjamini-Hochberg adjustment was performed across all analyses.

^g^Reference level.

^h^K6: Kessler-6 Psychological Distress Scale.

### Past-Year Psychotherapy Use

#### Descriptives and Demographics for Past-Year Psychotherapy Use

We found that about one-third (331/971, 34.1%) of the participants reported past-year psychotherapy use. Among sociodemographic characteristics, only educational attainment (*P*<.001) and sexual orientation (*P*=.04) had statistically significant relationships with past-year psychotherapy use (refer to Table 3 for full results). Higher psychological use distress severity was significantly associated with greater odds of endorsing past-year psychotherapy use (OR 1.07, 95% CI 1.03-1.1; *P*<.001).

**Table 3:** Logistic regression predicting past-year psychotherapy use^a^ from sociodemographic characteristics in adults with psychological distress (n=970^b^).

Variable			OR^c,d^ (95% CI)	Adjusted *P* value^d,e^	
Age			0.99 (0.98-1.01)	.45	
**Gender**					.27
		Men	—^f^		
		Women	1.24 (0.91-1.68)		
		Nonbinary, other identity, or undisclosed	2.13 (1.04-4.44)		
**Sexual orientation**					.58
		Straight	—		
		Gay or lesbian	1.95 (1.11-3.41)		
		Bisexual	1.82 (1.24-2.64)		
		Other or undisclosed	1.56 (0.77-3.13)		
**Race and ethnicity**					.04
		Non-Hispanic White	—		
		Asian	0.63 (0.36-1.06)		
		Hispanic	1.22 (0.77-1.91)		
		Non-Hispanic Black	0.99 (0.54-1.77)		
		Other or multiracial	0.90 (0.47-1.67)		
**Income (US $)**					.49
		<15,000	—		
		15,000-25,000	1.44 (0.89-2.33)		
		25,000-34,999	1.39 (0.90-2.14)		
		35,000-49,999	1.05 (0.69-1.61)		
		50,000-74,999	0.75 (0.50-1.13)		
		75,000-99,999	0.92 (0.61-1.37)		
		100,000-149,999	1.13 (0.76-1.66)		
		>150,000	1.14 (0.79-1.64)		
**Education**					<.001
	No college		—		
	Some college		2.72 (1.83-4.09)		
	Associate’s degree		0.88 (0.59-1.31)		
	Bachelor’s degree		1.41 (1.06-1.88)		
	Graduate degree		0.90 (0.62-1.31)		
Psychological distress (K6^g^: 0-24)			1.07 (1.03-1.10)	<.001	

^a^The reference level is denying past-year psychotherapy use, such that OR >1 reflects an association with endorsing past-year psychotherapy use.

^b^This model is based on 970 participants with complete demographic data because 1 participant had a missing value for income.

^c^OR: odds ratio.

^d^For categorical variables (all except age and psychological distress), the *P* value shown is an omnibus *P* value calculated across levels of the variable.

^e^Benjamini-Hochberg adjustment was performed across all analyses.

^f^Reference level.

^g^K6: Kessler-6 Psychological Distress Scale.

#### Interest in GSH by Past-Year Psychotherapy Use

Among those (n=640) who denied past-year psychotherapy use, 77.7% (n=497) were at least “somewhat interested” in GSH, 39.8% (n=255) were at least “moderately interested,” and 11.3% (n=72) were “very interested.” Nearly one-fifth (n=143, 22.3%) of the participants were “not at all interested.” By contrast, among those (n=331) who endorsed past-year psychotherapy use, 86.1% (n=285) were at least “somewhat interested” in GSH, 57.7% (n=191) were at least “moderately interested,” and 28.4% (n=94) were “very interested.” Only 13.9% (n=46) of these participants were “not at all interested.” The difference in GSH interest between groups was statistically significant, that is, those who had used psychotherapy in the past year were significantly more interested in GSH than those who had not (OR 2.38, 95% CI 1.86-3.06; *P*<.001). This effect was not accounted for by psychological distress severity, which was not a statistically significant predictor of GSH interest in this model (OR 1.02, 95% CI 0.99-1.04; *P*=.43). Refer to Multimedia Appendix 1 for complete descriptive statistics and Multimedia Appendix 2 for a visualization.

#### Self-Reported Likelihood of Using GSH by Past-Year Psychotherapy Use

Among those denying past-year psychotherapy use, approximately one-third (205/640, 32%) reported that they would be likely to complete at least one GSH session, whereas over half (170/331, 51.4%) of those endorsing past-year psychotherapy use reported that they would be likely to do so. This difference was statistically significant (OR 2.25, 95% CI 1.71-2.96; *P*<.001). This effect was not accounted for by psychological distress severity, which was not a statistically significant predictor of self-reported likelihood of GSH use in this model (OR 1, 95% CI 0.97-1.03; *P*=.92). Refer to Multimedia Appendix 1 for complete descriptive statistics and Multimedia Appendix 2 for a visualization.

### Barriers to Psychotherapy Access

#### Descriptives for Barriers to Psychotherapy Access

Approximately one-third (206/640, 32.2%) of the participants denied that they “might benefit” from psychotherapy (ie, no perceived need). When the subgroup that endorsed perceived need (434/640, 67.8%) was given the opportunity to select multiple contributing reasons for not using psychotherapy, the most commonly endorsed barrier was “issues with money or insurance” (323/434, 74.4% of those endorsing perceived need). “Didn’t know where to go or who to see” (230/434, 53%) and “too busy/not enough time” (190/434, 43.8%) were the second- and third-most commonly endorsed barriers in this subgroup (refer to Table 4 for frequencies of all barriers and Multimedia Appendix 2 for a visualization).

**Table 4:** Frequency of endorsement of all contributing barriers to past-year psychotherapy use in a checklist format among participants who endorsed perceived need for psychotherapy and denied past-year psychotherapy use (n=434), presented by barrier type.

All contributing barriers to past-year psychotherapy use by barrier type		Participants, n (%)^a^
**Structural**		
	Problems with money or insurance^b^	323 (74.4)
	Not enough time or too busy	190 (43.8)
	Need to stay home or cannot get transportation	123 (28.3)
	Could not get an appointment	53 (12.2)
**Attitudinal**		
	No perceived need for psychotherapy	—^c^
	Want to handle the problem alone	180 (41.5)
	Do not want to share private information	169 (38.9)
	Psychotherapy will not work	101 (23.3)
	Worried about what others might think	84 (19.4)
**Other**		
	Did not know who to see or where to go	230 (53.0)
	Care provider might be culturally insensitive	84 (19.4)
	Other reason	43 (9.9)
	The problem went away	30 (6.9)

^a^Participants were given the opportunity to select multiple barriers, such that individual participants may be counted several times in this table.

^b^Each barrier description is abbreviated from the original answer choice presented to participants. See Multimedia Appendix 1 for full text of each answer choice.

^c^Participants were queried about perceived need for treatment before being presented a checklist of other potential barriers to treatment access. Those who denied perceived need were not given the opportunity to select multiple barriers and therefore are excluded from this table.

#### Interest in GSH by Individual Access Barriers

The lack of perceived need for psychotherapy was significantly associated with lower interest in GSH (for endorsing relative to denying perceived need: OR 2.11, 95% CI 1.55-2.88; *P*<.001). This effect was not accounted for by the effect of psychological distress severity, which was not a statistically significant predictor of GSH interest in this model (OR 0.99, 95% CI 0.96-1.03; *P*=.83). None of the other individual barriers had a statistically significant univariate relationship with interest in GSH. Refer to Table 5 for results of all univariate models, Multimedia Appendix 1 for full descriptive statistics, and Multimedia Appendix 2 for a visualization.

**Table 5:** Results of univariate logistic regressions for outcomes (1) interest in guided self-help (GSH) and (2) self-reported likelihood of guided self-help, by endorsement of each self-reported barrier to past-year psychotherapy use in a sample of adults with psychological distress (n=640).

Barrier^a^	GSH interest			Self-reported likelihood of GSH use^b^	
	OR^c^ (95% CI)	Adjusted *P* value^d^	OR (95% CI)		Adjusted *P* value^d^
Psychotherapy will not work^e^	0.71 (0.47-1.06)	.28	0.99 (0.62-1.58)		.97
The problem went away	0.76 (0.4-1.44)	.59	0.93 (0.41-2)		.92
Want to handle the problem alone	0.84 (0.59-1.19)	.55	0.97 (0.65-1.45)		.92
Do not want to share private information	0.76 (0.54-1.08)	.35	0.93 (0.62-1.39)		.83
Worried about what others think	1.51 (0.97-2.36)	.23	1.19 (0.72-1.95)		.67
Did not know who to see or where to go	0.98 (0.7-1.38)	.92	0.88 (0.6-1.31)		.72
Not enough time or too busy	0.94 (0.67-1.33)	.83	1.08 (0.73-1.61)		.83
Problems with money or insurance	1.27 (0.85-1.91)	.47	0.93 (0.59-1.47)		.83
Need to stay home or cannot get transportation	1.52 (1.04-2.23)	.13	1.17 (0.76-1.81)		.67
Could not get an appointment	1.13 (0.66-1.92)	.83	1.39 (0.76-2.48)		.51
Care provider might be culturally insensitive	0.97 (0.63-1.5)	.92	1.05 (0.63-1.72)		.92
Other reason	0.75 (0.43-1.31)	.55	0.62 (0.29-1.22)		.43

^a^Participants were given the opportunity to choose multiple answer choices. Each row in the table represents 2 independent univariate regressions (1 for each GSH-related outcome), where the single predictor is binary endorsement of the barrier in column 1. The reference level for the predictor variable is nonendorsement, such that OR >1 reflects an association with endorsing the barrier.

^b^The reference level is denying likely GSH use, such that OR >1 reflects an association with endorsing likely GSH use.

^c^OR: odds ratio.

^d^Benjamini-Hochberg adjustment was performed across all analyses.

^e^Each barrier description is abbreviated from the original answer choice presented to participants. See Multimedia Appendix 1 for full text of each answer choice.

#### Self-Reported Likelihood of GSH Use by Individual Access Barriers

By contrast, the relationship between the lack of perceived need and self-reported likelihood of GSH use did not reach statistical significance after Benjamini-Hochberg adjustment (for endorsing relative to denying “benefit:” OR 1.51, 95% CI 1.05-2.2; *P*=.12). None of the other individual barriers had a statistically significant relationship with interest in GSH. Refer to Table 5 for results of all univariate models, Multimedia Appendix 1 for full descriptive statistics, and Multimedia Appendix 2 for a visualization.

### Primary Access Barriers and Barrier Type

#### Descriptives and Demographics for Primary Barriers and Primary Barrier Type

When participants endorsing perceived need selected their primary barrier to past-year psychotherapy use, “issues with money or insurance” (170/640, 26.6%) was most commonly endorsed. Therefore, among all participants, this was the second-most common primary barrier relative to the lack of perceived need (206/640, 32.2%). The frequency of these 2 primary barriers far exceeded the frequency of the third-most common primary barrier, “wanted to handle the problem alone,” which was endorsed as primary barrier by only 7.3% (47/640) of the participants.

When barriers were grouped, attitudinal primary barriers were reported by over half of the participants (336/640, 52.5% of those without past-year psychotherapy use) and were more common than structural primary barriers (244/640, 38.1%). The remaining 9.4% (60/640) of the participants’ primary barriers fell into the “ other” category (refer to Table 6 for frequencies of all primary barriers).

The only individual characteristic that was a statistically significant predictor of primary barrier type was gender (*P*=.002), such that women had lower odds of reporting an attitudinal primary barrier relative to men (OR 0.46, 95% CI 0.32-0.67; refer to Table 7 for full results).

**Table 6:** Frequency of self-reported primary barriers among all participants who denied past-year psychotherapy use (n=640), presented by barrier type.

Primary barrier to past-year psychotherapy use by barrier type			Participants, n (%)
**Structural**			
	Problems with money or insurance^a^	170 (27)	
	Not enough time or too busy	37 (5.8)	
	Need to stay home or cannot get transportation	19 (3.0)	
	Could not get an appointment	12 (1.9)	
	Total	238 (37.7)	
**Attitudinal**			
	No perceived need for psychotherapy	206 (32.2)	
	Want to handle the problem alone	47 (7.3)	
	Does not want to share private information	40 (6.3)	
	Psychotherapy will not work	23 (3.6)	
	Worried about what others might think	8 (1.3)	
	Total	324 (50.7)	
**Other**			
	Did not know who to see or where to go	37 (5.8)	
	Care provider might be culturally insensitive	10 (1.6)	
	Other reason	27 (4.2)	
	The problem went away	4 (0.6)	
	Total	78 (12.2)	

^a^Each barrier description is abbreviated from the original answer choice presented to participants. See Multimedia Appendix 1 for full text of each answer choice.

**Table 7:** Results of a multivariate multinomial regression model predicting type of primary barrier to past-year psychotherapy use by sociodemographic characteristics in adults with psychological distress (n=640).

Characteristics			Attitudinal^a^, OR^b^ (95% CI)		Other, OR (95% CI)		Adjusted *P* value^c,d^
Age			1.03 (1.01-1.04)		1.00 (0.97-1.03)		.03
**Gender**							.002
	Men	—^e^		—			
	Women	0.46 (0.32-0.67)		1.16 (0.60-2.21)			
	Nonbinary, other identity, or undisclosed	0.53 (0.17-1.65)		1.86 (0.32-10.9)			
**Sexual orientation**							.07
	Straight	—		—			
	Gay or lesbian	0.92 (0.41-2.10)		0.85 (0.22-3.31)			
	Bisexual	0.38 (0.22-0.66)		0.90 (0.41-1.96)			
	Other or undisclosed	1.79 (0.67-4.77)		0.94 (0.17-5.09)			
**Race and ethnicity**							.59
	Non-Hispanic White	—		—			
	Asian	2.09 (1.04-4.20)		1.54 (0.51-4.61)			
	Hispanic	1.24 (0.66-2.32)		1.67 (0.66-4.20)			
	Non-Hispanic Black	1.66 (0.80-3.47)		2.11 (0.67-6.59)			
	Other or multiracial	1.09 (0.48-2.44)		2.09 (0.68-6.43)			
**Income (US $)**							.047
	<15,000	—		—			
	15,000-25,000	3.51 (1.54-8.03)		3.78 (1.22-11.8)			
	25,000-34,999	2.51 (1.16-5.44)		3.22 (1.10-9.39)			
	35,000-49,999	1.74 (0.86-3.52)		1.85 (0.69-4.96)			
	50,000-74,999	2.19 (1.21-3.97)		1.34 (0.51-3.55)			
	75,000-99,999	1.75 (1.02-2.98)		1.92 (0.78-4.73)			
	100,000-149,999	1.53 (0.92-2.56)		2.89 (1.25-6.70)			
	>150,000	1.17 (0.75-1.83)		0.39 (0.16-0.96)			
**Education**							.43
	No college	—		—			
	Some college	0.68 (0.41-1.12)		0.97 (0.41-2.26)			
	Associate’s degree	1.09 (0.66-1.81)		1.13 (0.46-2.79)			
	Bachelor’s degree	0.97 (0.67-1.40)		0.73 (0.40-1.34)			
	Graduate degree	1.49 (0.94-2.38)		0.75 (0.31-1.77)			
Psychological distress (K6^f^: 0-24)			0.96 (0.91-1.00)		0.95 (0.89-1.02)		.27

^a^The reference level for the outcome variable is endorsement of a structural primary barrier.

^b^OR: odds ratio.

^c^*P* values are omnibus values across all levels of the outcome variable (ie, structural, attitudinal, and other). For categorical predictors, the *P* value shown is an omnibus *P* value calculated across all levels of the predictor variable.

^d^Benjamini-Hochberg adjustment was performed across all analyses.

^e^Reference level.

^f^K6: Kessler-6 Psychological Distress Scale.

#### Interest in GSH by Primary Barrier Type

Among the participants with an attitudinal primary barrier, 69.3% (233/336) were at least “somewhat interested” in GSH, whereas among those with a structural primary barrier, 87.3% (213/244) were at least “somewhat interested.” Approximately one-third (108/336, 32.1%) of those with an attitudinal primary barrier were at least “moderately interested” in GSH versus nearly half (118/244, 48.4%) of those with a structural primary barrier. Only 8.3% (28/336) of the participants with an attitudinal primary barrier were “very interested” in GSH versus 14.8% (36/244) of those with a structural primary barrier. Finally, less than one-third of the individuals with an attitudinal primary barrier reported that they were “not at all interested” in GSH (103/336, 30.7%) compared to 12.7% (31/244) of the individuals with a structural primary barrier. Refer to Multimedia Appendix 1 for full descriptive statistics and Multimedia Appendix 2 for a visualization. Overall, the effect of primary barrier type on GSH interest was statistically significant (*P*<.001), such that individuals who reported an attitudinal primary barrier were less interested in GSH than those who reported a structural primary barrier (OR 0.44, 95% CI 0.32-0.6). This effect was not accounted for by the effect of psychological distress severity, which was not a statistically significant predictor of GSH interest in this model (OR 0.99, 95% CI 0.96-1.02; *P*=.72). The effect of reporting an “other”-type primary barrier was not statistically significant (OR 0.93, 95% CI 0.56-1.55), relative to reporting an attitudinal primary barrier.

#### Self-Reported Likelihood of GSH Use by Primary Barrier Type

Whereas 37.3% (91/244) of those with a structural primary barrier reported that they would be likely to complete at least 1 GSH session, 26.8% (90/336) of the participants with an attitudinal primary barrier reported that they would be likely to do so. This difference met statistical significance at *P*<.05 (OR 0.61, 95% CI 0.43-0.87; *P*=.045). This effect was not accounted for by psychological distress severity, which was not a statistically significant predictor of self-reported likelihood of GSH use in this model (OR 0.99, 95% CI 0.95-1.03; *P*=.43). Among those endorsing a primary barrier in the “other” category, 40% (24/60) reported that they would be likely to complete at least 1 GSH session. The effect of endorsing an “other”-category primary barrier relative to a structural primary barrier was not statistically significant (OR 1.11, 95% CI 0.62-1.98; *P*=.83).

### Univariate Analyses for Income and Race

#### Race and Ethnicity

Our sample consisted of just under one-third (306/971, 31.5%) people of color: 6.5% (63/971) non-Hispanic Black, 10.8% (105/971) Hispanic, 8.8% (85/971) Asian, and 5.5% (53/971) other races or multiracial. See Multimedia Appendix 1 for full descriptive statistics for GSH interest and likely GSH use by racial and ethnic group. In a series of univariate analyses, race did not have a statistically significant relationship with each past-year psychotherapy use (ORs 0.53-1.26; *P*=.59), primary barrier type (*P*=.43; for attitudinal relative to structural, ORs 0.75-2.18; for other relative to structural, ORs 1.54-1.97), interest in GSH (ORs 0.72-1.23; *P*=.58), or self-reported likelihood of using GSH (ORs 0.56-1.07; *P*=.45).

#### Income

Income had a statistically significant relationship with primary barrier type at *P*<.05 (*P*=.045), but did not have a statistically significant relationship with each past-year psychotherapy use (ORs 0.54-1.44; *P*=.45), interest in GSH (ORs 0.66-1.3; *P*=.43), or self-reported likelihood of GSH use (ORs 0.63-1.6; *P*=.13). The pattern of the effect of income on primary barrier type was inconsistent across income levels. For example, compared to the group at the lowest income level (<US $15,000), individuals with slightly greater incomes had greater odds of reporting an attitudinal primary barrier (eg, US $15,000-US $24,999; OR 3.4, 95% CI 1.59-7.28), but individuals with much greater incomes did not significantly differ from individuals in the lowest income level (eg, >US $150,000; OR 1.05, 95% CI 0.7-1.59). Refer to Multimedia Appendix 1 for full descriptive statistics for GSH interest and likely GSH use by income level, and Multimedia Appendix 2 for a visualization.

## Discussion

### Principal Findings

In this study, we found reasons to question the extent to which DMHIs may reach individuals with unmet needs by circumventing structural barriers alone. In a sample of individuals with psychological distress, those who did not use psychotherapy in the past year were *less* interested in GSH and *less* likely to predict that they would use GSH relative to those who had used psychotherapy. This difference might be explained by the high prevalence of attitudinal (vs structural) barriers observed among those without past-year psychotherapy use, especially the lack of perceived treatment need. Attitudinal barriers were the most commonly reported barriers to past-year psychotherapy use and were associated with lower interest in GSH and lower self-reports of likely GSH use. Taken together, these results suggest the potentially 3 substantial role of these barriers in limiting DMHI uptake for individuals with unmet treatment needs. Interestingly, we did not find support for differences in any of the major study variables by racial-ethnic identification, and results for differences by income were mixed.

### Limitations

A limitation of this study is that our analyses are restricted to participants’ opinions drawn from a brief description of a single DMHI. Our results about an internet-delivered GSH bibliotherapy might not generalize to other DMHI formats if barriers to DMHI use vary across digital delivery formats [31]. For example, privacy could be a greater concern when using a smartphone app, and attitudes might differ when human guidance is not included [25,31].

Our sample also presents limits to generalizability. First, this study focused exclusively on US residents, which limits generalizability to other countries, for example, due to different health care systems [27]. In addition, because all participants were recruited online, it is possible that unique features of this population might alter our sample’s interest in DMHIs relative to the general population (eg, they may have higher digital literacy [75,76]). Future work extending this research to varying samples will be required to establish generalizability.

Our measurement methods may have introduced error. Self-reported likelihood of GSH use may overestimate actual likelihood of GSH use, and future research should measure observable behavior instead. Our measure of interest in GSH may also have lost sensitivity in its format as a 4-point scale [77]; however, Leung [78] presented a discussion of psychometric performance relative to 5-, 7-, and 11-point scales. Finally, our survey design also imposes some limitations on the interpretability of these data. Participants without a perceived need for psychotherapy were not queried about any other barriers, obscuring the possible role of multiple barriers for this group. We also queried participants about their experience of multiple specific barriers via a checklist-format question, which may distort results by encouraging participants to overendorse answer choices [48]. Therefore, this work might be stronger if measurement allowed participants to rate the relevance of each barrier dimensionally to capture greater complexity.

### Strengths

This study’s major novel contribution is its direct investigation of the relationship between perceived barriers to accessing “traditional” psychotherapy and potential DMHI use. Previous literature has extensively reported barriers to general mental health treatment seeking [25-28,79,80] and DMHI interest and adherence [81] separately but often neglected to measure them together. Barriers to DMHI acceptability and engagement have often been studied inductively, often among participants in clinical trials or institutional settings *already using* DMHIs [30,31]. By contrast, our approach offered a unique contribution to the literature by reflecting potential barriers to the *uptake* of DMHIs. First, we captured a wide range of both attitudinal and structural barriers, rather than focusing on only those commonly addressed in the DMHI literature (eg, cost and geography). This allowed us to capture common barriers to help seeking in general, derived from robust existing literature [25-28] rather than technology-specific concerns. In addition, we sampled from a general population of individuals with potential treatment needs rather than trial participants who have already “overcome” barriers to uptake.

Importantly, we avoided the common negligence to investigate racial and ethnic differences in DMHI research [33,39,45], placing special emphasis on both racial and ethnic minorities and low-income individuals. In addition, we designed our sample to focus on individuals with unmet treatment needs by (1) requiring at least mild psychological distress and (2) conducting primary analyses on the subsample that had not accessed psychotherapy.

Finally, another unique strength of this study is that we parsed participants’ *interest* in GSH from their self-reported likelihood of *actually using* GSH**.** The predictors of these outcomes were similar across analyses, but their differing base rates contributed unique information. For example, among individuals who did not access psychotherapy in the past year, the percentage of the participants who predicted that they would use GSH (205/640, 32%) was approximately half the percentage of the participants who were at least somewhat interested (497/640, 77.7%). According to the theory of planned behavior, participants’ estimates of their likely treatment-seeking behavior should be a stronger predictor of actual treatment use than attitudes [47,49,50]. Understanding factors that lead to differences in attitudes versus perceived likelihood of using DMHIs may be informative for dissemination efforts.

### Implications

Our results suggest that the attitudinal barriers that limit traditional psychotherapy use may also limit DMHI use. Individuals who are not in treatment due to the lack of perceived need for *any* treatment are likely to forgo use of DMHIs. Our results were also consistent with previous literature by suggesting that attitudinal barriers to psychotherapy use may be more common than the structural barriers that DMHIs may target [25,27]. Failing to address attitudinal barriers may substantially limit the potential public health impact of DMHIs if this obstacle is left unaddressed.

In addition, our results suggest that individuals who *already have access to* psychotherapy may be *more* likely to use DMHIs than those with unmet needs. This is concerning because a major aim of DMHIs is to reach individuals who cannot access traditional treatments. However, this finding does not contradict all models of DMHI dissemination. For example, DMHIs may improve access by expanding the spectrum of care intensities available [7]. If individuals with lower-intensity needs rely on lower-intensity treatments, they may free up “higher-intensity” resources (eg, specialized clinicians) for others. Whereas institutional systems of stepped care enforce this model (eg, Improving Access to Psychological Therapies program of the United Kingdom [13]), the public’s use of stand-alone DMHIs may not always follow this pattern. The hope that individuals using publicly available DMHIs (eg, commercially available apps [9]) might then forgo traditional treatment is only a hypothesis. Our results suggest that the contrary is possible, such that, individuals with positive treatment attitudes may use *both* DMHIs *and* traditional treatment. This would risk widening, rather than reducing, the treatment gap by benefiting those with existing access rather than those without it. However, stand-alone DMHIs may have particular public health utility when they offer services that are difficult to access in the community (eg, specialty treatments such as cognitive behavior psychotherapy for insomnia [82]).

We did not find evidence supporting racial and ethnic differences in our study’s indicators of potential DMHI use, supporting the broad appeal of DMHIs. This is consistent with previous literature, which has generally found little support for differences in interest and engagement in DMHIs across racial and ethnic groups, although research is still limited [33]. We also did not find racial-ethnic differences in the relative rate of attitudinal versus structural barriers to treatment access. This contrasts with previous findings that attitudinal barriers to general help seeking may vary by race [41,83], especially stigma [84]. However, results are inconsistent and suggest that these patterns may be nuanced across individual minority groups and by treatment type (eg, medications vs psychological treatment [34]). In addition, we found some evidence for differences in attitudinal barriers and GSH interest by income, but the results were mixed, such that it is difficult to interpret a pattern. Future research should clarify the potentially complex relationship between income, access barriers, and help seeking [36].

Finally, our findings have implications for DMHI development research. DMHI trials may primarily attract participants who are *already interested* in psychological treatment but unable to access traditional psychotherapy due to structural barriers. Therefore, these samples might underrepresent individuals facing attitudinal barriers to treatment. This biased sample would not reflect the total target population of individuals with unmet needs. Therefore, the estimates of DMHI use could be inflated, and design elements intended to increase DMHI use might not be generalizable to the total population. This would be particularly concerning because the rates of DMHI use are already low, and much work has been devoted to testing the effectiveness of design alterations intended to increase engagement [81].

### Future Directions

Future work should first substantiate this exploratory research by investigating these questions in additional samples [60]. Recruiting dedicated samples of racial and ethnic minorities may also be key to developing a clearer picture of this phenomenon (eg, capturing intersectional effects). The lack of representation of racial-ethnic minorities is a well-documented problem in DMHI research [8,33,34,39,45], so this should be prioritized in future DMHI work. In addition, evidence suggests that attitudinal barriers may vary across countries [27], and treatment-seeking pathways vary across countries according to their health care systems [85]. Therefore, research with international samples and replication across research groups in several countries is essential for broad applicability and public health impact on a global scale. Finally, community-based sampling may improve upon potential generalizability and data integrity issues introduced by online recruitment [62]. Community-based sampling might also facilitate improved measurement approaches, such as observed uptake of clinical services, rather than relying on self-report.

In addition, future research should seek to better understand the lack of perceived need for treatment. Our results and previous literature [26] suggest that a lack of perceived need may be the most common reason individuals do not access treatment. Therefore, using measurement approaches that are able to parse the components of perceived need (and lack thereof) may be particularly informative. For example, low mental health literacy [86] may contribute to a lack of perceived need when an individual does not have sufficient understanding of mental health concerns to recognize their symptoms. In addition, reporting a lack of perceived need for treatment may reflect a cultural preference for other types of help, such as informal community support [87]. Developing a more nuanced understanding of perceived need may be the first step toward understanding how lacking perceived need for treatment might generalize to DMHIs as well as how perceived need might differ across treatment modalities.

Future work should also endeavor to synthesize barriers to general treatment seeking with findings about barriers to DMHIs specifically. Indeed, both barriers to help seeking in general [25,29] and barriers specific to DMHIs [30,31] cumulatively serve as bottlenecks in DMHIs’ ability to reach individuals with unmet needs. Therefore, attempts to ameliorate these issues and expand the reach of DMHIs will need to address both types of barriers. In addition, individuals may sometimes seek treatment *despite* attitudinal barriers [88]. Therefore, in addition to interventions aimed at modifying attitudes, future research might also seek to promote factors that circumvent perceived barriers. For example, research informed by the theory of planned behavior [47] suggests that subjective social norms and perceived behavioral control predict treatment-seeking behavior [48,49].

Finally, future work might pay greater attention to existing approaches to increasing mental health treatment seeking. Help-seeking interventions [89,90] are often designed to target barriers such as stigma [79] and mental health literacy [91]. In addition to encouraging general treatment seeking, interventions could be tailored to encourage the use of DMHIs. For example, models for “direct-to-consumer marketing” of evidence-based psychological interventions have been proposed as an adjunct to treatment innovation and implementation efforts [92]. Tailoring such interventions to build public awareness of the diverse “portfolio” of treatment options [7] available for their direct access [93] could potentially increase the reach of DMHIs. Raising awareness of DMHIs’ existence and efficacy [89] could empower treatment seekers to choose the options that are most fitting for their attitudes and preferences [86].

### Conclusions

Overall, this work suggests the importance of questioning assumptions about how potential solutions to the mental health treatment gap will reach those who most need them. DMHIs clearly have an advantage over traditional psychotherapy in their scalability because their low resource requirements and low intensity of use can circumvent structural barriers. However, understanding the wide range of obstacles to DMHIs’ dissemination and uptake may be essential to maximizing their impact. In the quest to expand treatment access, the *first* hurdle that DMHIs face is the willingness to seek mental health treatment *of any kind.* Our work suggests that addressing individuals’ attitudinal barriers may be an important step to ensure that DMHIs maximally achieve their promise of expanding access to treatment and reducing the public health burden of psychopathology. Devoting resources to help-seeking interventions [89] may be key to addressing the treatment gap [6] rather than focusing on developing solutions to structural barriers alone (ie, scalable treatments). Importantly, adequate representation of the underserved groups most in need of accessible interventions is vital throughout this research [8,33].

## Appendix

Multimedia Appendix 1 Supplementary tables.

Multimedia Appendix 2 Supplementary figures.

## Abbreviations

ACT: acceptance and commitment therapy
DMHI: digital mental health intervention
DWM: Doing What Matters in Times of Stress
GSH: guided self-help
K6: Kessler-6 Psychological Distress Scale
MTurk: Amazon Mechanical Turk
OR: odds ratio
WHO: World Health Organization

## Data Availability

The dataset generated and analyzed during this study is available in the Open Science Framework repository online [74].
