# Supplementary material for: Psychotherapy Access Barriers and Interest in Digital Mental Health Interventions Among Adults With Treatment Needs: Survey Study
Source: JMIR Ment Health. 2025 Apr 1;12:e65356. doi: 10.2196/65356 (PMC12000781; doi:10.2196/65356)
Supplement: Multimedia Appendix 1 [file mental_v12i1e65356_app1.docx]

**Multimedia Appendix 1 – Supplementary Tables**

Supplementary Table 1. Full text of answer choices shown to participants for barriers to past-year psychotherapy use.

| Abbreviation used in manuscript tables | Full text of answer choice |
| --- | --- |
| Psychotherapy will not work | Didn’t think it would work |
| The problem went away | The problem went away by itself |
| Want to handle the problem alone | I wanted to handle the problem on my own |
| Do not want to share private information | Didn’t want to share private information about myself |
| Worried about what others think | I was worried about what others might think if they found out |
| Did not know who to see or where to go | Didn’t know where to go or who to see |
| Not enough time or too busy | Too busy / not enough time |
| Problems with money or insurance | Issues with money or health insurance |
| Need to stay home or cannot get transportation | Problems like lack of transportation, couldn’t find a babysitter, need to stay home to take care of somebody |
| Could not get an appointment | Couldn’t get an appointment (therapists/doctors said they were full or put me on a waiting list) |
| Care provider might be culturally insensitive | The professional might be insensitive about (or wouldn’t understand) my race, culture, identity, or something similar |
| Other reason | Other reason (please write below): |

Supplementary Table 1. Self-Reported Likelihood of Using Guided Self-Help by Past-Year Psychotherapy Use.

| **Characteristic** |  | **No, N = 206^1^** | **Yes, N = 434^1^** |
| --- | --- | --- | --- |
| GSH_interest |  |  |  |
| Not at all |  | 67 (33%) | 76 (18%) |
| Somewhat |  | 78 (38%) | 164 (38%) |
| Moderately |  | 48 (23%) | 135 (31%) |
| Very |  | 13 (6.3%) | 59 (14%) |
| Likely GSH Use |  | 54 (26%) | 151 (35%) |
|  | ^1^n (%) | | |

Supplementary Table 2. Interest in Guided Self-Help and Self-Reported Likelihood of Guided Self-Help Use by Type of Primary Barrier to Psychotherapy Access.

| **Characteristic** | **Structural, N = 244^1^** | **Attitudinal, N = 336^1^** | **Other, N = 60^1^** |
| --- | --- | --- | --- |
| GSH_interest |  |  |  |
| Primary Barrier Type | 31 (13%) | 103 (31%) | 9 (15%) |
| Somewhat | 95 (39%) | 125 (37%) | 22 (37%) |
| Moderately | 82 (34%) | 80 (24%) | 21 (35%) |
| Very | 36 (15%) | 28 (8.3%) | 8 (13%) |
| Likely GSH Use | 91 (37%) | 90 (27%) | 24 (40%) |
| ^1^n (%) | | | |

Supplementary Table 3. Interest in Guided Self-Help by Endorsement of Each Contributing Barrier to Psychotherapy Access for Participants Who Endorsed Perceived Need for Psychotherapy.

| **Characteristic** | **Not at all, N = 76^1^** | **Somewhat, N = 164^1^** | **Moderately, N = 135^1^** | **Very, N = 59^1^** |
| --- | --- | --- | --- | --- |
| It won't work |  |  |  |  |
| Not endorsed | 57 (75%) | 117 (71%) | 113 (84%) | 46 (78%) |
| Endorsed | 19 (25%) | 47 (29%) | 22 (16%) | 13 (22%) |
| Problem went away |  |  |  |  |
| Not endorsed | 72 (95%) | 148 (90%) | 127 (94%) | 57 (97%) |
| Endorsed | 4 (5.3%) | 16 (9.8%) | 8 (5.9%) | 2 (3.4%) |
| Handle it alone |  |  |  |  |
| Not endorsed | 43 (57%) | 94 (57%) | 77 (57%) | 40 (68%) |
| Endorsed | 33 (43%) | 70 (43%) | 58 (43%) | 19 (32%) |
| Sharing private info |  |  |  |  |
| Not endorsed | 47 (62%) | 90 (55%) | 87 (64%) | 41 (69%) |
| Endorsed | 29 (38%) | 74 (45%) | 48 (36%) | 18 (31%) |
| Others might think |  |  |  |  |
| Not endorsed | 65 (86%) | 131 (80%) | 116 (86%) | 38 (64%) |
| Endorsed | 11 (14%) | 33 (20%) | 19 (14%) | 21 (36%) |
| Didn't know where |  |  |  |  |
| Not endorsed | 40 (53%) | 66 (40%) | 74 (55%) | 24 (41%) |
| Endorsed | 36 (47%) | 98 (60%) | 61 (45%) | 35 (59%) |
| Too busy |  |  |  |  |
| Not endorsed | 48 (63%) | 81 (49%) | 80 (59%) | 35 (59%) |
| Endorsed | 28 (37%) | 83 (51%) | 55 (41%) | 24 (41%) |
| Money/insurance |  |  |  |  |
| Not endorsed | 29 (38%) | 33 (20%) | 34 (25%) | 15 (25%) |
| Endorsed | 47 (62%) | 131 (80%) | 101 (75%) | 44 (75%) |
| Stay home/need transport |  |  |  |  |
| Not endorsed | 59 (78%) | 122 (74%) | 93 (69%) | 37 (63%) |
| Endorsed | 17 (22%) | 42 (26%) | 42 (31%) | 22 (37%) |
| Waitlisted |  |  |  |  |
| Not endorsed | 67 (88%) | 144 (88%) | 121 (90%) | 49 (83%) |
| Endorsed | 9 (12%) | 20 (12%) | 14 (10%) | 10 (17%) |
| Cultural insensitivity |  |  |  |  |
| Not endorsed | 63 (83%) | 126 (77%) | 117 (87%) | 44 (75%) |
| Endorsed | 13 (17%) | 38 (23%) | 18 (13%) | 15 (25%) |
| Other reason |  |  |  |  |
| Not endorsed | 70 (92%) | 141 (86%) | 125 (93%) | 55 (93%) |
| Endorsed | 6 (7.9%) | 23 (14%) | 10 (7.4%) | 4 (6.8%) |
| ^1^n (%) | | | | |

Supplementary Table 4. Descriptive Statistics for Self-Reported Likelihood of Using Guided Self-Help by Endorsement of Each Contributing Barrier to Psychotherapy Access for Participants Who Endorsed Perceived Need for Psychotherapy.

| **Characteristic** | **No, N = 283^1^** | **Yes, N = 151^1^** |
| --- | --- | --- |
| It won't work |  |  |
| Not endorsed | 217 (77%) | 116 (77%) |
| Endorsed | 66 (23%) | 35 (23%) |
| Problem went away |  |  |
| Not endorsed | 263 (93%) | 141 (93%) |
| Endorsed | 20 (7.1%) | 10 (6.6%) |
| Handle it alone |  |  |
| Not endorsed | 165 (58%) | 89 (59%) |
| Endorsed | 118 (42%) | 62 (41%) |
| Sharing private info |  |  |
| Not endorsed | 171 (60%) | 94 (62%) |
| Endorsed | 112 (40%) | 57 (38%) |
| Others might think |  |  |
| Not endorsed | 231 (82%) | 119 (79%) |
| Endorsed | 52 (18%) | 32 (21%) |
| Didn't know where |  |  |
| Not endorsed | 130 (46%) | 74 (49%) |
| Endorsed | 153 (54%) | 77 (51%) |
| Too busy |  |  |
| Not endorsed | 161 (57%) | 83 (55%) |
| Endorsed | 122 (43%) | 68 (45%) |
| Money/insurance |  |  |
| Not endorsed | 71 (25%) | 40 (26%) |
| Endorsed | 212 (75%) | 111 (74%) |
| Stay home/need transport |  |  |
| Not endorsed | 206 (73%) | 105 (70%) |
| Endorsed | 77 (27%) | 46 (30%) |
| Waitlisted |  |  |
| Not endorsed | 252 (89%) | 129 (85%) |
| Endorsed | 31 (11%) | 22 (15%) |
| Cultural insensitivity |  |  |
| Not endorsed | 229 (81%) | 121 (80%) |
| Endorsed | 54 (19%) | 30 (20%) |
| Other reason |  |  |
| Not endorsed | 251 (89%) | 140 (93%) |
| Endorsed | 32 (11%) | 11 (7.3%) |
| ^1^n (%) | | |

Supplementary Table 5. Interest in Guided Self-Help and Self-Reported Likelihood of Guided Self-Help Use by Income.

| **Characteristic** | **< $15000**  **N=1221** | **$15000-$25000**  **N = 1011** | **$25000-$34999**  **N = 1161** | **$35000-$49999**  **N = 1451** | **$50000-$74999**  **N = 1911** | **$75000-$99999**  **N = 1221** | **$100000-$149999**  **N = 1111** | **$150000+**  **N = 621** |
| --- | --- | --- | --- | --- | --- | --- | --- | --- |
| GSH Interest |  |  |  |  |  |  |  |  |
| Not at all | 26 (21%) | 21 (21%) | 22 (19%) | 27 (19%) | 40 (21%) | 18 (15%) | 19 (17%) | 16 (26%) |
| Somewhat | 43 (35%) | 27 (27%) | 31 (27%) | 57 (39%) | 70 (37%) | 37 (30%) | 38 (34%) | 20 (32%) |
| Moderately | 31 (25%) | 33 (33%) | 38 (33%) | 43 (30%) | 50 (26%) | 44 (36%) | 32 (29%) | 21 (34%) |
| Very | 22 (18%) | 20 (20%) | 25 (22%) | 18 (12%) | 31 (16%) | 23 (19%) | 22 (20%) | 5 (8.1%) |
| Likely GSH Use | 37 (30%) | 41 (41%) | 55 (47%) | 45 (31%) | 73 (38%) | 58 (48%) | 42 (38%) | 24 (39%) |
| 1n (%) | | | | | | | | |

Supplementary Table 6. Interest in Guided Self-Help and Self-Reported Likelihood of Guided Self-Help Use by Race.

| **Characteristic** | **Non-Hispanic White, N = 665^1^** | **Non-Hispanic Black, N = 63^1^** | **Hispanic, N = 105^1^** | **Asian, N = 85^1^** | **Other or Multiracial, N = 53^1^** |
| --- | --- | --- | --- | --- | --- |
| GSH Interest |  |  |  |  |  |
| Not at all | 133 (20%) | 7 (11%) | 22 (21%) | 18 (21%) | 9 (17%) |
| Somewhat | 224 (34%) | 25 (40%) | 23 (22%) | 34 (40%) | 18 (34%) |
| Moderately | 180 (27%) | 25 (40%) | 40 (38%) | 26 (31%) | 21 (40%) |
| Very | 128 (19%) | 6 (9.5%) | 20 (19%) | 7 (8.2%) | 5 (9.4%) |
| Likely GSH Use | 265 (40%) | 23 (37%) | 42 (40%) | 23 (27%) | 22 (42%) |
| ^1^n (%) | | | | | |
