# Supplementary material for: Psychotherapy Access Barriers and Interest in Digital Mental Health Interventions Among Adults With Treatment Needs: Survey Study
Source: JMIR Ment Health. 2025 Apr 1;12:e65356. doi: 10.2196/65356 (PMC12000781; doi:10.2196/65356)
Supplement: Multimedia Appendix 2 [file mental_v12i1e65356_app2.docx]

**Multimedia Appendix 1 – Supplementary Figures**

Supplementary Figure 1.

Self-reported barriers to past-year psychotherapy use sorted by frequency and categorized by barrier type.


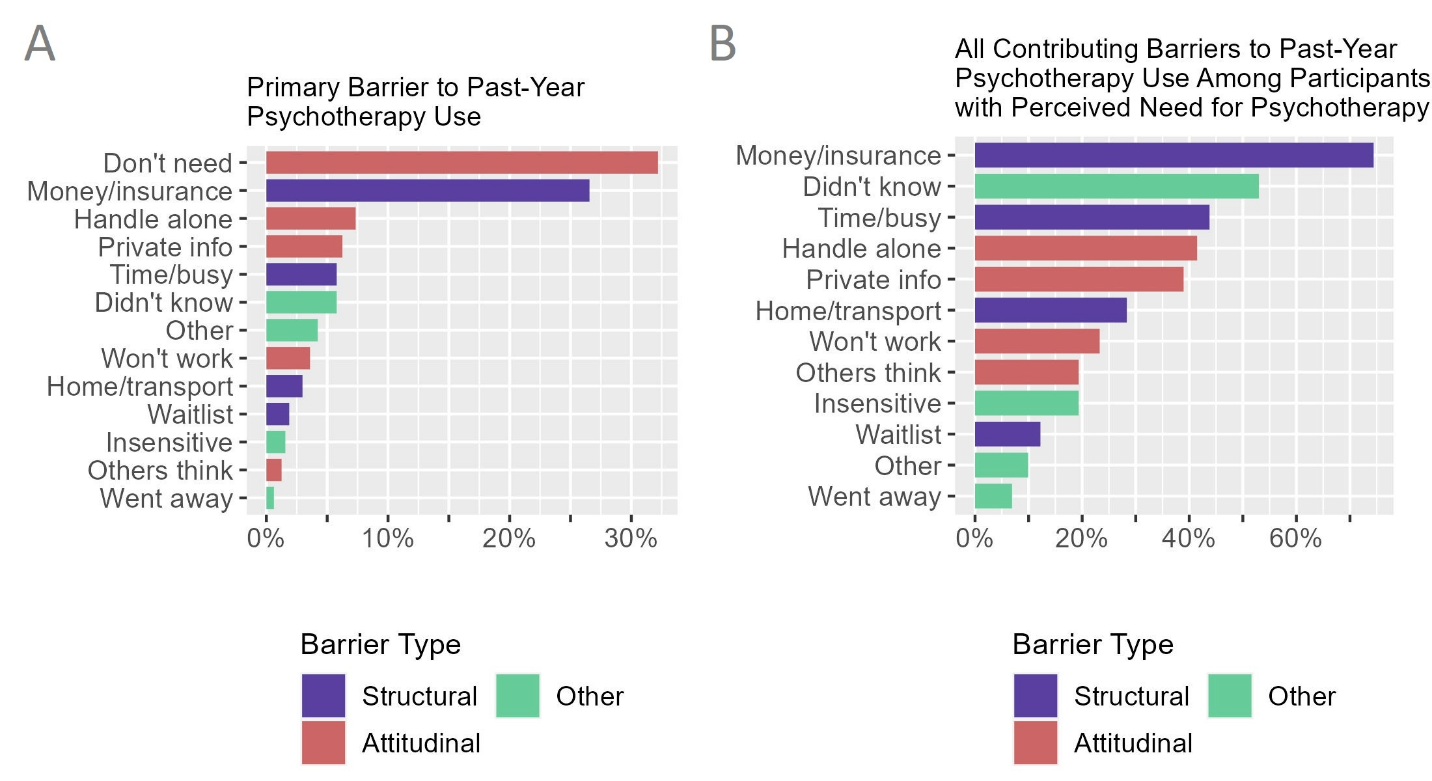


Note. Panel A reflects data for all participants who reported no past-year psychotherapy use (n=640). Panel B reflects data for only those participants who both reported no past-year psychotherapy use and endorsed perceived need for psychotherapy in the past year (n=434). Abbreviations: GSH, guided self-help

Supplementary Figure 2.

Primary Outcomes (Interest in Guided Self-Help and Self-Reported Likelihood of Guided Self-Help Use) by Income and Past-Year Psychotherapy Use in Adult Participants with at Least Moderate Psychological Distress


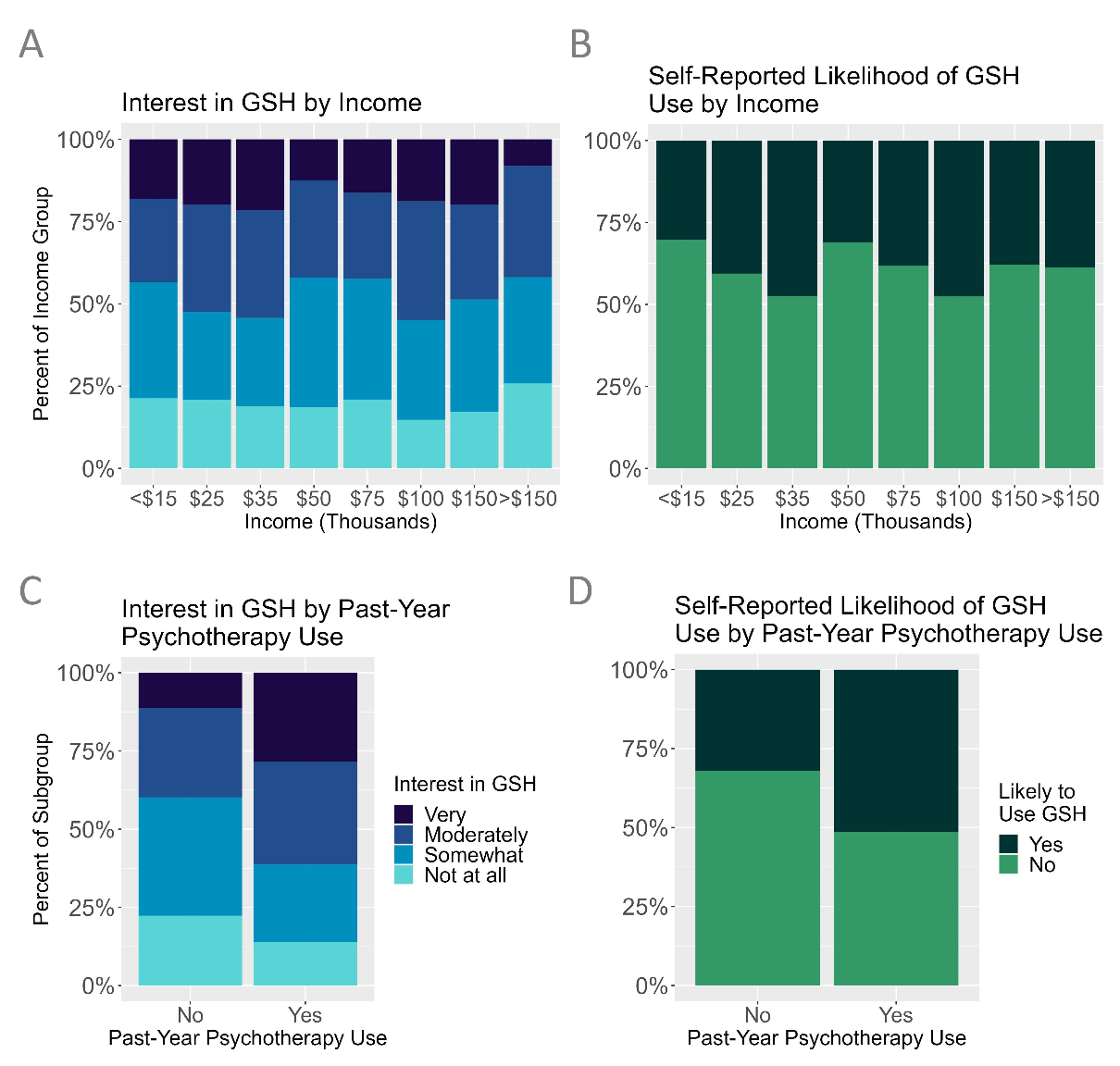


Note. For panels A-B, N=970, and for panels C-D, N=971 (complete sample), due to missing income value for 1 participant. For panels A-B, x-axis labels reflect the top of each income range (except for “>$150). Abbreviations: GSH, guided self-help.

Supplementary Figure 3. Interest in Guided Self-Help and Self-Reported Likelihood of Using Guided Self-Help by Self-Reported Barriers to Psychotherapy Access in the Past Year: Type of Primary Barrier and Each Contributing Barrier


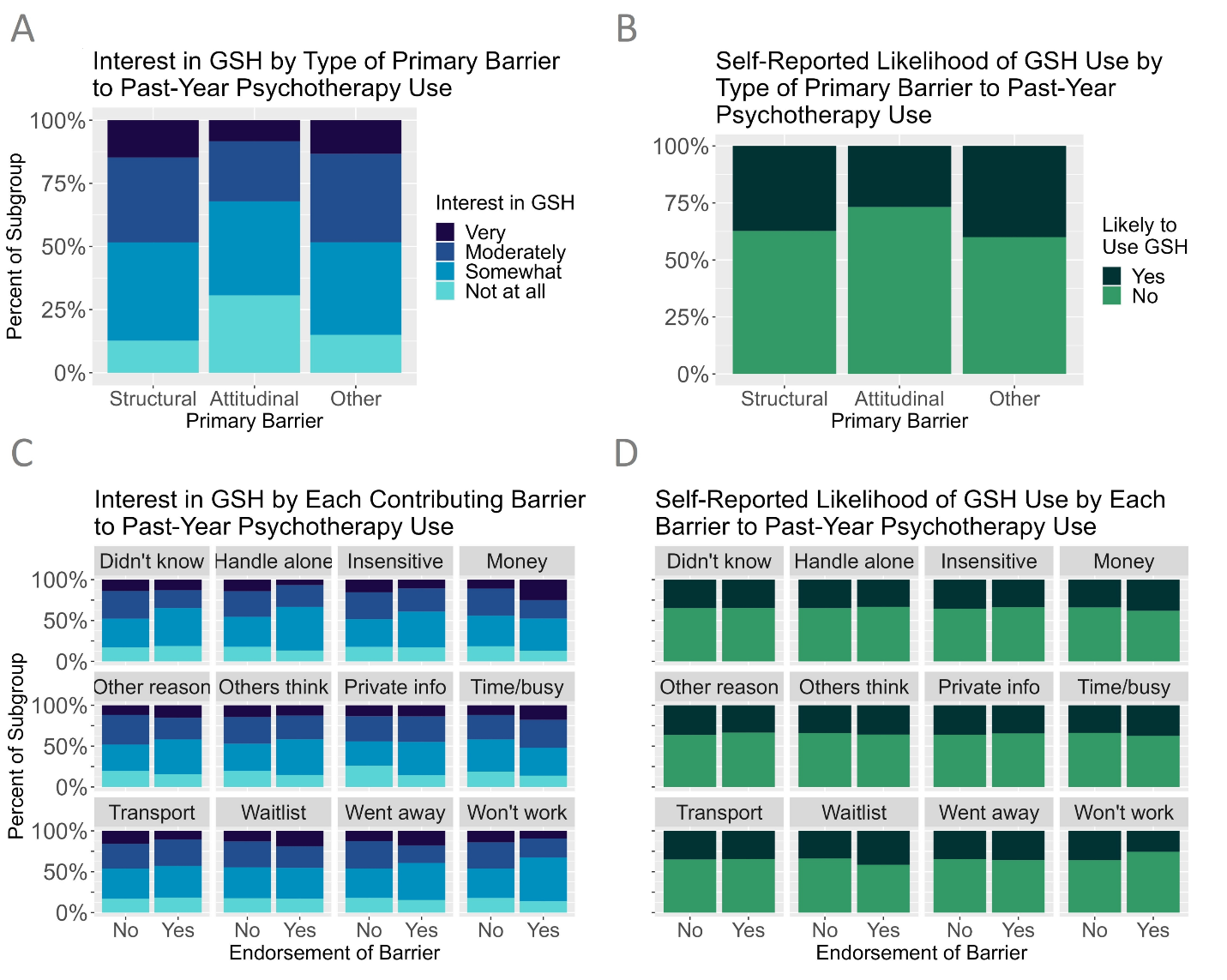
Note. Panes A and B reflect data for all participants who reported no past-year psychotherapy use (n=640). Panes C and D reflect data for only those participants who both reported no past-year psychotherapy use and endorsed perceived need for psychotherapy in the past year (n=434). Abbreviations: GSH, guided self-help
